# Ependymomas in infancy: underlying genetic alterations, histological features, and clinical outcome

**DOI:** 10.1007/s00381-020-04655-x

**Published:** 2020-05-30

**Authors:** Stephanie T. Jünger, Felipe Andreiuolo, Martin Mynarek, Evelyn Dörner, Anja zur Mühlen, Stefan Rutkowski, Andre O. von Bueren, Torsten Pietsch

**Affiliations:** 1 Department of Neuropathology, DGNN Brain Tumor Reference Center, University of Bonn, Bonn, Germany; 2 Department of Neurosurgery, University of Cologne Medical Center, Cologne, Germany; 3 Department of Pediatric Hematology/Oncology, Medical Center Hamburg-Eppendorf, Hamburg, Germany; 4 Division of Pediatric Hematology and Oncology, Department of Pediatrics, Obstetrics and Gynecology, University Hospital of Geneva, Geneva, Switzerland

**Keywords:** Ependymoma, Infant, Genetics, Neuropathology, Fusion genes

## Abstract

**Introduction:**

Young age is an adverse prognostic factor in children with ependymomas. Treatment of these infants is challenging since beneficial therapeutic options are limited. As ependymomas are considered a biologically heterogeneous group, we aimed to characterize infant ependymomas with regard to their histological and genetic features.

**Materials and methods:**

We analyzed 28 ependymomas occurring in children younger than 18 months at diagnosis enrolled into the HIT2000-E protocols with the aim to postpone irradiation until the age of 18 months if possible. All cases underwent neuropathological review, including immunohistochemical characterization. Genome-wide copy number alterations (CNA) were assessed by molecular inversion probe assays, and *RELA* and *YAP1* fusions were detected by RT-PCR and sequencing.

**Results:**

All infant ependymomas were anaplastic (WHO grade III). Twenty-one (75%) cases were located in the posterior fossa. Gross total resection was accomplished in 12 (57%) of these cases. All posterior fossa tumors showed loss of H3-K27me^3^ characteristic of PFA ependymomas. CNA analysis showed a stable genome in all cases with lack of chromosome 1q gain, an adverse prognostic marker in PFA ependymomas of older children. However, after a median follow-up of 5.4 years, 15 (71%) relapsed, and 9 (43%) died. Seven ependymomas (25%) occurred in the supratentorial region. Gross total resection could be achieved in only two of these cases. Four tumors carried *C11orf95-RELA* fusions, and two cases had typical *YAP1-MAMLD1* fusions (one case was not analyzable). The *RELA*-fused cases did not display *CDKN2A* loss as an adverse indicator of prognosis in this disease entity. Although three infants (43%) with supratentorial ependymomas relapsed, all patients survived (median follow-up, 8.0 years).

**Conclusion:**

Infant ependymomas seem to fall into three biological entities, with supratentorial tumors carrying *RELA* or *YAP* fusions and PFA posterior fossa ependymomas. The latter showed a poor outcome even though chromosome 1q gain was absent.

## Introduction

Ependymomas are the third most common brain tumor in children [1]. They can arise along the entire neuroaxis. In children, most tumors are located intracranially and two thirds of them in the posterior fossa (PF) [2]. Despite histological resemblance, ependymomas represent distinct disease entities with different cells of origin, genetic pathways activated in their pathogenesis, epigenetic landscape, and clinical behavior [3, 4]. The usefulness of the WHO grading system [5] for risk stratification is controversial [6–11]. Despite major progress in the understanding of the biology of ependymomas, therapeutic options remain limited and mainly include maximal safe surgery and radiation therapy (RT) [7, 8]. Previous trials proved that chemotherapy administered as monotherapy is ineffective [12], but the possible role of chemotherapy is not fully resolved yet and is currently being tested in the European SIOP EP-II trial [7, 11].

Furthermore, the AIEOP trial as well as recently published data by Merchant et al. showed evidence that some patients may be cured by surgery alone [7, 9]. So far, risk stratification as a basis for risk-adapted therapy mainly relies on clinical parameters including age at diagnosis, residual tumor after surgery, and local versus metastatic disease at presentation. In addition, some protocols also differentiate according to location (supratentorial versus infratentorial) and histological grading [7–9].

Although improved risk models have been proposed in the past dividing tumors according to their cytogenetic patterns into numerical, balanced, or structural subgroups [13], evaluating the presence of individual prognostic factors such as gain of chromosome 1q in PF ependymoma [2, 9, 13, 14], *RELA* and *YAP1* fusions in supratentorial (ST) ependymomas [3, 15–18], or analyzing specific epigenetic patterns [3, 19], none of these have yet been included in stratification of patients enrolled in clinical trials.

Most published risk models include children with ependymomas of all ages, locations, and underlying genetics ignoring the differences in biology and treatment among individual age groups. The group of infants with PF ependymoma apparently faces worst prognosis; at the same time, this group seems to be the one most difficult to treat in terms of surgery and adjuvant treatment [20].

In this study, we analyzed a total of 28 patients under the age of 18 months at the time of diagnosis (retrieved from a cohort of 203 pediatric ependymoma), treated according to the HIT ependymoma trial, with the majority harboring PF ependymomas, focusing on their clinical, histological, and genetic particularities.

## Material and methods

### Patients

Ependymomas in 28 children under the age of 18 months were retrieved from the archives of the Institute of Neuropathology, University of Bonn Medical Center (Bonn, Germany), and of the DGNN German Brain Tumor Reference Center (Bonn, Germany). All patients were diagnosed with anaplastic ependymoma (WHO grade III) in a period from 2002 to 2011 and enrolled into the prospective non-randomized multicenter HIT ependymoma study (ClinicalTrials.gov NCT00303810). This included risk-adapted HIT-SKK chemotherapy (without intraventricular methotrexate) and irradiation. In infant patients, the aim was to postpone irradiation until the age of 18 months if possible. In case of residual tumor, patients were evaluated for potential second-look surgery after each therapy element. All examinations were carried out on the basis and according to the legal requirements of the revised Declaration of Helsinki of the World Medical Association in 1983 and according to the guidelines of the institutional review boards. Informed consent was given at study inclusion by the parents.

### Histopathology and immunohistochemistry

All tumors were centrally reviewed at the DGNN German Brain Tumor Reference Center (Bonn, Germany) and reclassified according to the revised WHO classification of tumors of the CNS 2016 [5]. Mitotic activity was assessed in 4μm-thick HE-stained FFPE slides by counting mitotic figures in 10 high power fields (HPF; area, 2.38 mm^2^). Presence of more than 10 mitoses/10 HPF was defined as high mitotic activity. Further histological features including necrosis, vascular proliferation, and clear cell morphology were assessed. Immunohistochemical staining—performed on an immunostaining system (BenchMark XT, Ventana-Roche, Mannheim, Germany)—included Ki67 (MAb MIB-1, Dako, Glostrup, Denmark), phosphohistone-H3 (Biocare, Concord, USA), p16 protein (MAb E6H4, Ventana-Roche, Darmstadt, Germany), p65-RelA (rabbit antibody D14E12, Cell Signaling, Danvers, U.S.A.), and trimethylated histone3-Lys27 (H3-K27me^3^; rabbit MAb C36B11, Cell Signaling). For H3-K37me^3^, positive nuclei of endothelial cells served as internal control.

### DNA extraction and copy number analysis

On hematoxylin and eosin (HE)-stained FFPE sections, representative areas with at least 80% tumor cell content were identified for microdissection and DNA extraction from serial sections using the QIAamp DNA Mini Kit (Qiagen, Hilden, Germany) according to the manufacturer’s instructions. In order to identify genome-wide copy number alterations and allelic disbalances, we used molecular inversion probe (MIP) assays (OncoScan FFPE express 330 K arrays, versions 2 and 3; Affymetrix, Santa Clara, CA, US) as previously described [21, 22]. Raw data were further analyzed with the Nexus Copy Number software, version 7.5 (BioDiscovery, El Segundo, CA, USA). DNA from FFPE cerebellar tissue served as normal control. The SNP-FASST-2 segmentation algorithm was used for data processing. All cases were reviewed individually, and if necessary, diploidy correction was performed manually.

### RNA extraction and detection of *C11orf95-RELA and YAP1-MAMLD1* fusions

FFPE or fresh frozen tissue (if available) of supratentorial ependymomas was further tested for presence of *C11orf95-RELA* and *YAP1-MAMLD1* fusions. RNA was extracted, followed by RT-PCR and sequencing, described previously [15, 16, 18].

### Statistical analyses

EFS and OS data was shown in Kaplan-Meier plots applying log-rank (Mantel-Cox) method using IBM SPSS statistics version 23 (IBM, Bonn, Germany). Results with a *p* value < 0.05 were regarded as significant.

## Results

### Supratentorial ependymomas

**Fig. 1:**
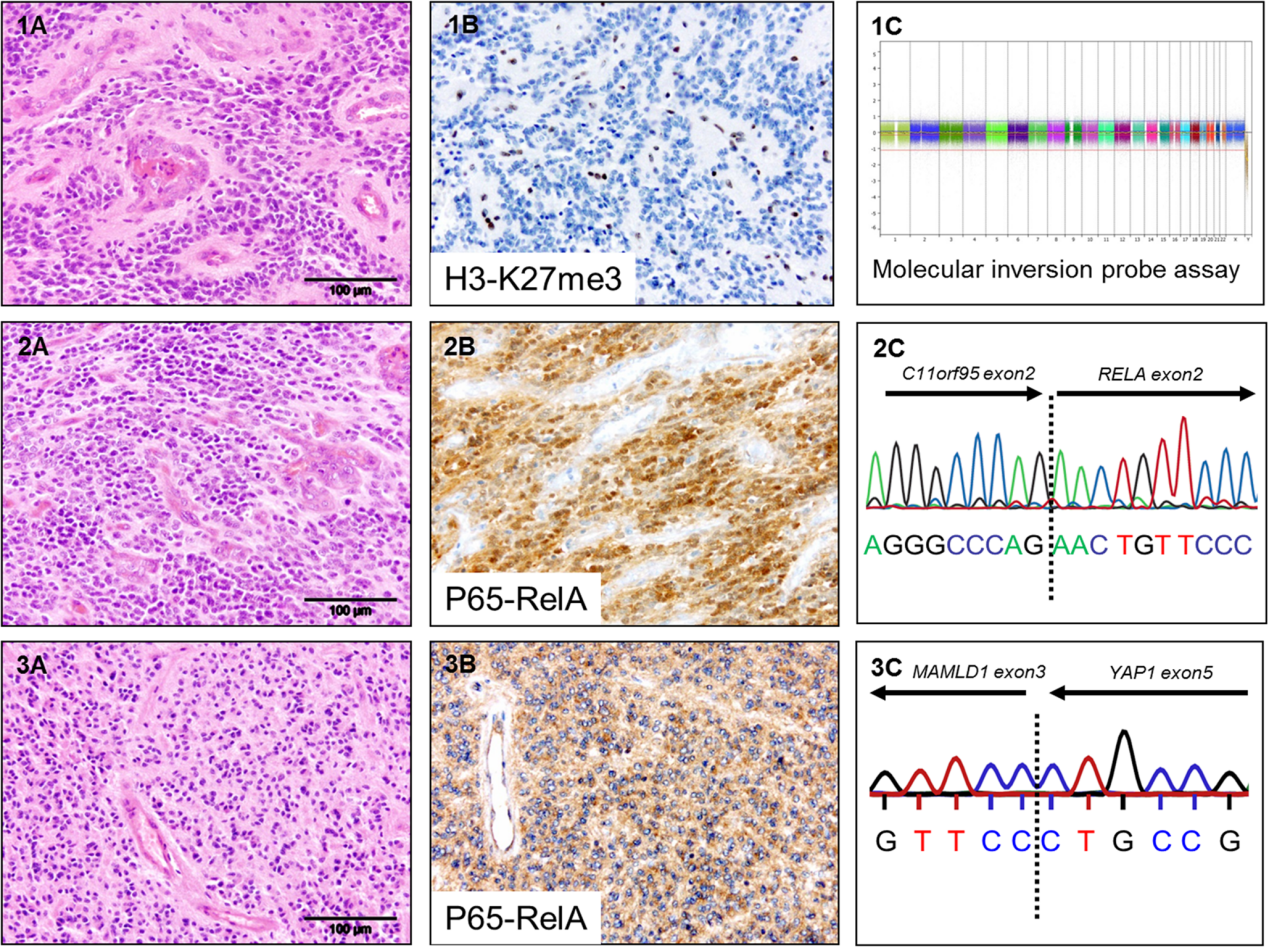
Histological and genetic features. **1** PFA ependymoma: (A) H&E, (B) H3-K27me^3^ immunohistochemistry indicating complete loss in tumor cells, and (C) CNV plot showing a balanced genome (MIP). **2** RELA ependymoma: (A) H&E, (B) nuclear p65-RelA accumulation, and (C) cDNA sequencing of *C11orf95-RELA* fusion transcript. **3** YAP ependymoma: (A) H&E, (B) cytoplasmatic p65-RelA staining, and (C) cDNA sequencing of *YAP1-MAMLD1* fusion transcript

Seven patients of 28 (25%) had an ependymoma in a supratentorial location. These occurred in five girls and two boys. Subtotal resection (STR) was performed in five (71%), and in three (43%), a relapse occurred. However, all patients were alive after a median follow-up of 7.96 years (range 0.28–12 years). High mitotic activity, necrosis, and vascular proliferation each were present in all but one case. All cases had at least one of these criteria. All tumors were classified as anaplastic ependymoma (WHO grade III). Two cases showed clear cell morphology, and balanced genomes were found in four cases (57%) compared to structural (two cases) and numerical genomic group (one case). Chromosome 1q gain was not present at all, while one case showed chromosome 22 loss. Six cases could be studied for fusion transcripts; for the remaining one, material for further analysis was not available. *RELA* fusions were detected in four cases, which showed nuclear accumulation of p65-RelA protein as indicator of activation of NFkB signaling. Two of the *RELA*-fused cases displayed clear cell morphology, and three of the four relapsed. *YAP1*-*MAMLD1* fusions were found in two other cases, not carrying *RELA* fusions. Concerning the latter two patients, none relapsed, although in one, gross total resection (GTR) could not be achieved. One of the two cases harboring a *YAP1*-*MAMLD1* fusion showed monosomy of chromosome 22. *CDKN2A* loss/LOH or p16 protein loss was not identified in any of these 7 cases. For further information, see Table 1 and Fig. 1.

**Table 1:** Clinical, histological, and genetic characteristics

	All locations		Supratentorial		Posterior fosssa	
Number of cases	28	%	7	%	21	%
Female	15	54	5	71	10	48
Male	13	46	2	29	11	52
Dead	9	32	0	0	9	43
Alive	19	68	7	100	12	57
Age at diagnosis : Mean	0.93 y		0.78 y		0.97 y	
Median	0.95 y		0.89 y		1.05 y	
5-year EFS		46		57		43
5-year OS		82		100		71
Subtotal resection	14	50	5	71	9	43
Initial metastatic disease	1	4	1	14	0	0
Relapse	18	64	3	43	15	71
WHO grade II	0	0	0	0	0	0
WHO grade III	28	100	7	100	21	100
Necrosis	24	86	6	86	18	86
Mitotic Activity > 10 mitoses/ 10 HPF	21	75	6	86	15	71
Vascular proliferation	25	89	6	86	19	90
True ependymal rosettes	0	0	0	0	0	0
Clear cell morphology	2	7	2	29	0	0
Genomic group: Numerical	1	4	1	14	0	0
Balanced	25	89	4	57	21	100
Structural	2	7	2	29	0	0
Chromosome 1q gain	0	0	0	0	0	0
Chromosome 22 loss	1	4	1	14	n.a.	
*C11orf95-RELA* fusion	4	14	4	57	n.a.	
*CDKN2A* loss	0	0	0	0	n.a.	
*YAP1-MAMLD1* fusion	2	7	2	29	n.a.

### Posterior fossa ependymomas

Twenty-one of 28 patients (75%) developed tumors in the posterior fossa. The male to female ratio was even (11 vs. 10). All cases were classified as anaplastic ependymoma (WHO grade III). In terms of histological criteria associated with anaplasia, 19 (91%) revealed vascular proliferation, 18 (86%) necrosis, and 15 (71%) high mitotic activity, while neither true ependymal rosettes nor clear cell morphology were encountered. Most strikingly, all cases showed a balanced genomic profile with consequently no gain of chromosome 1q and loss of the trimethylated lysine 27 of histone 3, typical of pediatric “group A” PF ependymomas [23, 24] (Table 1, Fig. 1).

Nine patients (43%) had postoperative residual tumor, and 15 (71%) suffered a relapse; none showed initial metastasis. After a median follow-up time of 5.44 years, 5-year EFS and OS were 43% and 71%, respectively.

### Statistical analysis

**Fig. 2:**
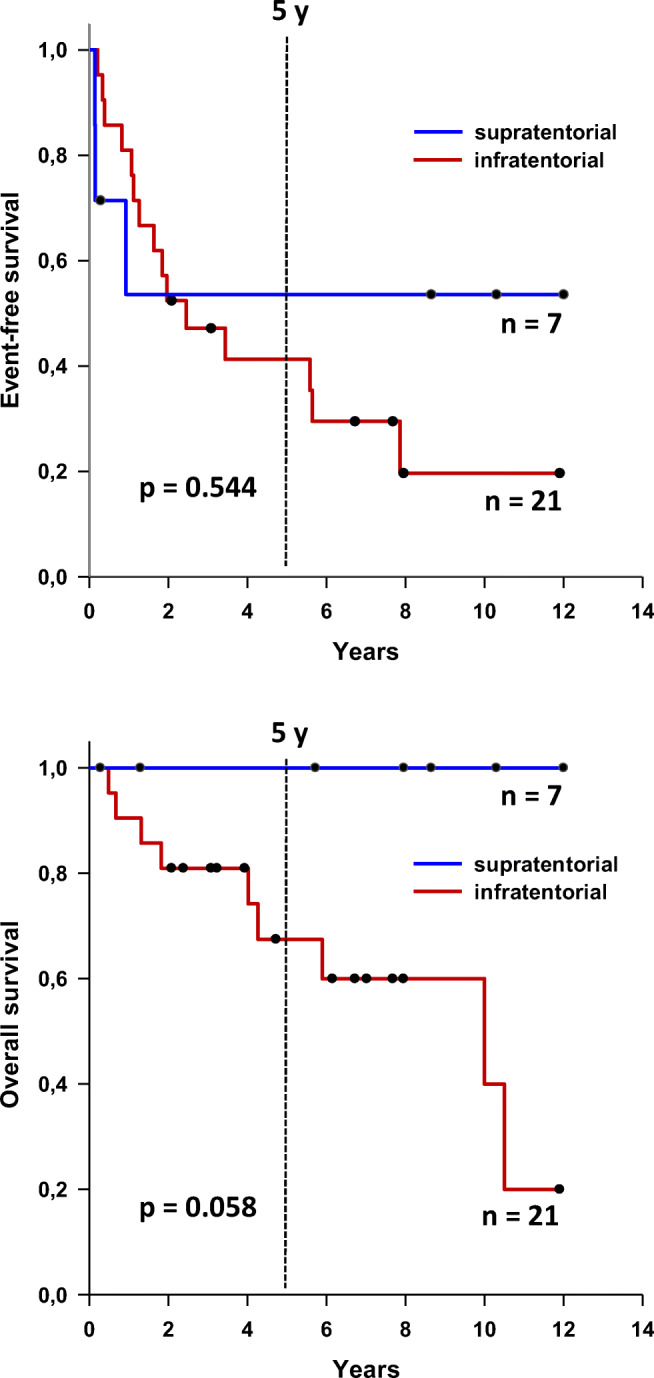
Survival analysis comparing ST and PF ependymoma below 18 months

Kaplan-Meier analysis revealed no statistically significant difference between ST and PF ependymoma regarding EFS and OS, respectively. Regarding OS, there was nevertheless a clear trend towards a favorable prognosis of ST ependymoma of infancy, since none of the patients died (Fig. 2).

## Discussion

Our results show that ependymomas of infancy (0–18 months) comprise distinct location- and age-associated ependymoma entities in terms of underlying biology and genetics and—probably resulting from the latter—their prognosis. In contrast to previous publications, we considered ST and PF ependymomas as different entities and therefore analyzed the populations separately with regard to clinical, histological, and genetic parameters.

Until now, a separate analysis according to infant age and tumor location was not provided in most published clinical trials. In addition, neuropathological features and genetic characteristics were usually not described in detail. However, Upadhyaya et al. recently published their prospective study for children below the age of 3 years analyzing tumors of different locations individually regarding prognostically relevant factors [11].

In our study, the period of recruitment (10 years) was comparingly short, and patients were enrolled into the risk-adapted HIT trial ruling out major population inhomogeneity. Due to case selection and specific characterization mentioned above, the number of cases—remaining from a large cohort of pediatric ependymoma (*n* = 203)—unfortunately was limited.

### Supratentorial ependymoma in infants show specific fusion transcripts and favorable overall survival

Strikingly, no patient with a supratentorial ependymoma below the age of 18 months at diagnosis died during the period of follow-up although all tumors in this age group were graded as anaplastic (WHO grade III). *RELA* fusions—representing the most frequent alteration in pediatric supratentorial ependymomas—were present in four cases (57%), which is similar in frequency as previously reported [3, 15, 17]. All three genomic groups are represented in tumors of this location. However, in line with Upadhyaya et al., we cannot confirm the poor prognosis reported by Pajtler et al. in a retrospective case collection [3, 11]. Although some patients relapsed, they were successfully treated, either with second resection, chemotherapy, or radiotherapy. One explanation for a favorable prognosis of this specific subgroup may be the absence of *CDKN2A* loss/LOH in *RELA*-positive tumors. *CDKN2A* loss has been previously reported in ST ependymoma and is known to be an indicator of adverse prognosis in *RELA*-fused ependymomas [25] and other brain tumors such as *IDH*-mutated anaplastic astrocytoma [26].

In addition, in this location, tumors harboring a *YAP1*-*MAMLD1* fusion—apparently a parameter of favorable prognosis [3, 11, 15, 17]—accounted for two patients (29%), and neither of them suffered from relapse or died.

### Posterior fossa ependymoma in infants show a balanced genome and frequent recurrence

It is generally accepted that patients with PF ependymomas face a worse prognosis, especially at young age; however, a recent publication showed no age-dependent prognostic difference for these tumors [9]. Although radiation therapy can be effective in infants, most neurooncological centers are reluctant to apply irradiation in this age group because of severe long-term secondary effects. Therefore, the treatment of infants imposes difficulties compared to older patients with PF ependymoma; hence, there is a need for identification of specific risk factors and novel treatment options in this subgroup of patients.

The rate of incomplete resection, a parameter of inferior prognosis, occurred in 43% of infratentorial compared to 71% in supratentorial ependymoma of infancy. The shorter overall survival of patients with posterior fossa ependymoma may result from re-resection being more difficult compared to the supratentorial compartment.

All PF ependymomas in this study were classified as anaplastic (WHO grade III). The prognostic impact of WHO grading was demonstrated in some studies but not found in others [9, 11].

Furthermore, genomic analysis revealed that all 21 patients in this group showed balanced genomic profiles and were confirmed to represent PF-A group/CIMP + ependymoma, immunohistochemistry demonstrating a complete loss of H3K27me^3^, which represents the subgroup of ependymoma with the worst prognosis [3, 27]. Interestingly, chromosome 1q gain and the associated structural genomic profile—both reported indicators of adverse prognosis [2, 5, 13, 14, 28, 29]—were not found in any of the 21 PF tumor samples in our cohort.

### Ependymomas of infancy represent distinct tumor entities and impose distinct challenges in terms of clinical management

In summary, we were able to demonstrate that ependymomas of infancy represent distinct location- and age-associated ependymoma entities in terms of underlying biology and genetics. Even though statistical significance regarding survival could not be reached, most probably due to the small cohort sizes, ST ependymomas seem to have a superior prognosis compared to PF ependymoma in terms of OS. The EFS of patients with tumors of the two locations may not be different, but re-resection being more easily achieved in the ST-compartment may lead to the long-term survival benefits noted in the previous studies.

ST ependymomas harbor distinct genetic alterations (such as fusions) which represent possible therapeutic targets; these fusions are absent in PF ependymomas. Besides the optimal timing for radiation therapy, the field of targeted therapies remains to be explored for ependymoma in the future.

Our results show that risk stratification for pediatric ependymoma remains challenging and suggest that separate analysis—regarding risk factors and treatment regimens—is required for tumors in different locations and among different age groups.

## Data Availability

The data that support the findings of this study are available on reasonable request from the corresponding author.
